# Switching Electrolyte Interfacial Model to Engineer Solid Electrolyte Interface for Fast Charging and Wide‐Temperature Lithium‐Ion Batteries

**DOI:** 10.1002/advs.202201893

**Published:** 2022-07-17

**Authors:** Gang Liu, Zhen Cao, Peng Wang, Zheng Ma, Yeguo Zou, Qujiang Sun, Haoran Cheng, Luigi Cavallo, Shiyou Li, Qian Li, Jun Ming

**Affiliations:** ^1^ State Key Laboratory of Rare Earth Resource Utilization Changchun Institute of Applied Chemistry Chinese Academy of Sciences Changchun 130022 P. R. China; ^2^ University of Science and Technology of China Hefei 230026 P. R. China; ^3^ Physical Science and Engineering Division (PSE) King Abdullah University of Science and Technology (KAUST) Thuwal 23955‐6900 Saudi Arabia; ^4^ School of Petrochemical Technology Lanzhou University of Technology Lanzhou 730050 P. R. China

**Keywords:** electrolyte solvation structure, fast charging, lithium‐ion batteries, solid electrolyte interfaces, wide‐temperature

## Abstract

Engineering the solid electrolyte interphase (SEI) that forms on the electrode is crucial for achieving high performance in metal‐ion batteries. However, the mechanism of SEI formation resulting from electrolyte decomposition is not fully understood at the molecular scale. Herein, a new strategy of switching electrolyte to tune SEI properties is presented, by which a unique and thinner SEI can be pre‐formed on the graphite electrode first in an ether‐based electrolyte, and then the as‐designed graphite electrode can demonstrate extremely high‐rate capabilities in a carbonate‐based electrolyte, enabling the design of fast‐charging and wide‐temperature lithium‐ion batteries (e.g., graphite | LiNi_0.6_Co_0.2_Mn_0.2_O_2_ (NCM622)). A molecular interfacial model involving the conformations and electrochemical stabilities of the Li^+^‐solvent‐anion complex is presented to elucidate the differences in SEI formation between ether‐based and carbonate‐based electrolytes, then interpreting the reason for the obtained higher rate performances. This innovative concept combines the advantages of different electrolytes into one battery system. It is believed that the switching strategy and understanding of the SEI formation mechanism opens a new avenue to design SEI, which is universal for pursuing more versatile battery systems with greater stability.

## Graphical abstract

A new concept of switching electrolyte interfacial model is presented to tune the solid electrolyte interphase (SEI) properties, by which a specific thinner SEI is pre‐formed on graphite electrode in ether‐based electrolyte first and then such SEI coated electrode (i.e., graphite@SEI) can be applied in the commercial carbonate‐based electrolyte to achieve a fast‐charging and wide‐temperature lithium‐ion battery.

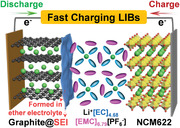

## 1 Introduction

Rechargeable metal‐ion batteries (e.g., Li^+^, Na^+^) have dominated the energy market from portable electronic devices to electric vehicles (EVs), in which the solid electrolyte interphase (SEI) chemistry has attracted great attention in the past two decades.^[^ ^1^ ^]^ This is because the SEI can be always formed on the electrode surface by electrolyte decomposition in the initial cycles, while such as‐formed SEI can protect the electrode and mitigate further electrolyte decomposition, in turn enabling better electrode stability and higher battery performance.^[^ ^2^ ^]^ Currently, many approaches, including the electrode coating (e.g., atomic layer deposition,^[^ ^3^ ^]^ molecular layer deposition,^[^ ^4^ ^]^ polymer surface coating,^[^ ^5^ ^]^ etc.) and electrolyte engineering^[^ ^6^ ^]^ have been widely developed to control the compositions and structures of SEI to pursue better performances.^[^ ^7^ ^]^ Among them, the electrolyte engineering, such as changing electrolyte compositions^[^ ^8^ ^]^ or adding film‐forming additives (e.g., vinylene carbonate (VC),^[^ ^9^ ^]^ vinyl ethylene carbonate (VEC),^[^ ^10^ ^]^ vinyl ethylene sulfite (VES),^[^ ^10^ ^]^ ethylene sulfate (DTD)^[^ ^11^ ^]^, etc.), has become the most effective strategy to tune SEI in practical applications. In this way, many unique SEI or CEI can be formed on the anode (e.g., graphite,^[^ ^12^ ^]^ Si/C‐based anode^[^ ^13^ ^]^) and the cathode (e.g., LiNi_1‐x‐y_Co_x_Mn_y_O_2_,^[^ ^14^ ^]^ high voltage cathode^[^ ^15^ ^]^) in the lithium‐ion batteries (LIBs), boosting the stability and reliability of LIBs significantly. Moreover, the same strategies can be also applied to construct the SEI on lithium metal and other electrodes in lithium batteries,^[^ ^16^, ^17^ ^]^ as well as other kinds of metal‐ion batteries (e.g., Na^+^,^[^ ^1c^ ^]^ K^+^,^[^ ^18^ ^]^ Zn^2+^,^[^ ^19^ ^]^ etc.). However, it remains a challenge to interpret the SEI formation mechanisms and their effect on different electrolytes, as the electrolyte decompositions follow diverse principles depending on the electrolyte compositions and electrode properties.

Herein, we introduce a completely new strategy of switching electrolyte to tune the SEI properties, endowing the electrode with a specific SEI first in one particular electrolyte which can show much better battery performance in another more general electrolyte. As a paradigm, we discover that a thinner SEI can be formed on the graphite anode (i.e., graphite@SEI) in an ether‐based electrolyte, then such as‐obtained graphite@SEI can be applied in the commercial carbonate‐based electrolyte, enabling the construction of a fast‐charging LIBs readily. Besides, we construct an interfacial model to demonstrate the differences in electrolyte decomposition paths from the ether‐based to the carbonate‐based electrolyte, in which the conformation and electrochemical stabilities of the Li^+^‐solvent‐anion complex were demonstrated to be the main factor affecting the SEI formation mechanisms, thereby elucidating the reason for the obtained higher rate performance. This new approach differs from those of electrode coating and/or electrolyte engineering reported before, by which we can combine the advantages of different electrolytes into one battery system readily. We hope our strategy could open a new avenue to design SEI and is universal for the design of more versatile battery systems with greater stability.

## 2 Results and Discussion

### 2.1 Concept of Switching Electrolyte Interfacial Model

**Figure 1:**
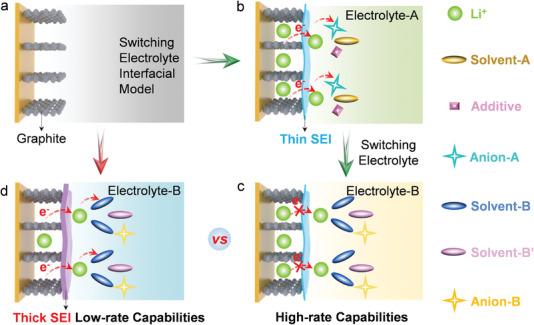
Constructing artificial SEI on electrodes. a) Pristine graphite electrode. b) Pre‐form a thin SEI on the graphite electrode in electrolyte‐A, and then c) such a thin SEI‐coated graphite electrode demonstrates high‐rate capabilities in electrolyte‐B. d) A thick SEI was formed on the pristine graphite electrode in electrolyte‐B, showing low‐rate capabilities.

We present a new concept of switching electrolyte interfacial model to tune SEI properties, by which the electrode was cycled in one specific electrolyte first to form a unique SEI and then such SEI coated electrode (i.e., electrode@SEI) was used in another targeted electrolyte to achieve better performances. In a paradigm case illustrated in **Figure** **1**a–c, a thin SEI that pre‐formed on the electrode (graphite) in the specific electrolyte (i.e., electrolyte‐A) can demonstrate better performance in the targeted electrolyte (i.e., electrolyte‐B), such as the high‐rate performance. In contrast, such high performance cannot be achieved when the pristine electrode (graphite) was cycled directly in the targeted electrolyte (Figure 1d). This idea is inspired by the fact that different SEI can be formed from the electrolyte decomposition in different electrolytes with distinct electrolyte interfacial models, which can show different effects on battery performances depending on their specific properties. Thus, this strategy can combine the different advantages of different electrolytes into one battery system readily. We demonstrate the innovative points of our strategy with the commonly used ether‐based and carbonate‐based electrolytes as an empirical example.

**Figure 2:**
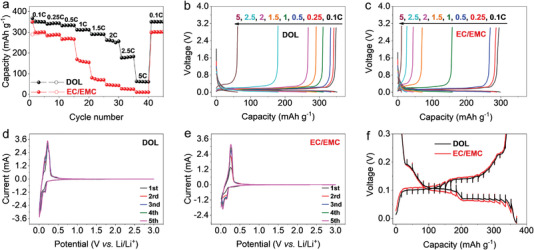
Electrochemical performance of graphite. a) Comparative rate capabilities and b,c) Voltage versus capacity profile, d,e) CV, and f) GITT curves of graphite electrode that operated in ether‐based electrolyte versus carbonate‐based electrolyte.

First of all, we notice significantly different lithium storage capabilities of graphite electrode in the ether‐based and carbonate‐based electrolytes. For example, an extremely high‐rate capacity can be obtained in the ether‐based electrolyte containing 1.0 m lithium bis(trifluoromethane sulfonyl)imide (LiTFSI) and 0.4 m LiNO_3_ in dioxolane (DOL). An average capacity of 352, 343, 334, 312, 291, 258, 180, and 62 mAh g^−1^ can be delivered at the rate of 0.1, 0.25, 0.5, 1, 1.5, 2, 2.5, and 5 C, respectively. These values are much higher than 296, 287, 268, 160, 74, 45, 26, and 11 mAh g^−1^ obtained in the commercial carbonate‐based electrolyte of 1.0 m lithium hexafluorophosphate (LiPF_6_) in ethylene carbonate/ethyl methyl carbonate (EC/EMC, v/v = 3/7) (**Figure** **2**a). Note that this is the first time reporting such high‐rate capabilities in an ether‐based electrolyte, as most ether‐based electrolytes are incompatible with graphite due to the graphite exfoliation caused by the Li^+^‐solvent co‐insertion.^[^ ^20^ ^]^ This is a breakthrough to design an ether‐based electrolyte with a normal concentration (i.e., 1.0 m) that is compatible with the graphite, where there is no need for employing a high‐concentration strategy.^[^ ^21^ ^]^ Herein, adding the DME solvent can make the electrolyte become incompatible with the graphite (i.e., 1.0 m LiTFSI, 0.4 m LiNO_3_ in DOL/DME) (Figure S1a, Supporting Information), and also the high rate capabilities cannot be obtained in the absence of LiNO_3_ (i.e., 1.0 m LiTFSI in DOL) (Figure S1b,c, Supporting Information). The reason for these phenomena will be discussed later. Besides, a much lower polarization is spotted in the ether‐based electrolyte, as judged by the comparative (dis‐)charge curves under different rates (Figure 2b,c, Supporting Information). The overlapped cyclic voltammetry (CV) curves further demonstrate better cycling stability of the graphite electrode in the ether‐based electrolyte (Figure 2d) compared to that in the carbonate‐based electrolyte (Figure 2e). The higher rate capabilities and lower polarization in the ether‐based electrolyte might result from a thinner SEI formed on the graphite electrode that has a higher Li^+^ transportation capability. The galvanostatic intermittent titration technique (GITT) results in Figure 2f also corroborate this viewpoint. It was found that the average Li^+^ diffusion coefficient (*D*_Li+_) within the graphite electrode is 3.91 × 10^−9^ cm^2^ s^−1^ in the ether‐based electrolyte, higher than 2.15 × 10^−9^ cm^2^ s^−1^ in the commercial carbonate‐based electrolyte during the lithiation states (Figure S2, Supporting Information).

**Figure 3:**
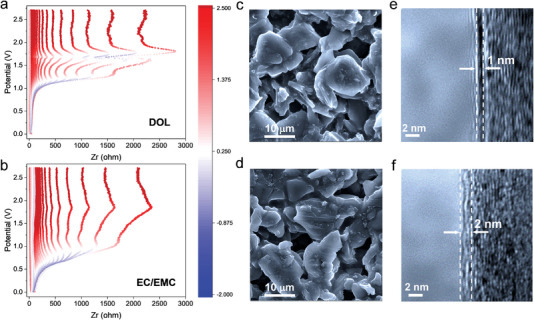
Characterizations of SEI. In situ EIS (top views of Nyquist plots) by discharging the half‐cell of lithium | graphite, SEM, and TEM images of the cycled graphite in the different electrolytes of a,c,e) ether‐based and b,d,f) carbonate‐based electrolyte.

The difference in SEI formed on the graphite electrodes was investigated by in situ electrochemical impedance spectroscopy (EIS) to interpret the different graphite performances.^[^ ^22^ ^]^ **Figure** **3**a,b shows the top view of the Nyquist plots acquired with different electrolyte systems, where the horizontal red‐white‐blue gradient band represents the slope change of the tangent line of the continuous data points of the normal semi‐circle. Specifically, from red to blue, the slope value changes from positive to negative, corresponding to the left side to the right side of the semi‐circle, and a broader gradient represents a larger radius of the semicircle, indicating a higher impedance. It is evident from the enlarged region where the graphite electrode in the ether‐based electrolyte system exhibits a lower impedance than that in the carbonate‐based electrolyte system (Figure S3a,b, Supporting Information). According to the fitted result in Figure S4 (Supporting Information), the impedance of the SEI (i.e., *R*_SEI_) formed in the ether‐based electrolyte is 20.84 Ω, which is much lower than 78.55 Ω in the carbonate‐based electrolyte. This result is in good accordance with the GITT test that the graphite electrode with SEI formed in ether‐based electrolyte allows faster Li^+^ transportation. In addition, the SEM in Figure 3c,d and TEM images in Figure 3e,f confirm a smoother and thinner SEI formed on the graphite electrode in the ether‐based electrolyte. In detail, the thickness of SEI formed in the ether‐based electrolyte is about 1.0 nm, while the thickness of SEI formed in the carbonate‐based electrolyte is about 2.0 nm. The thinner SEI presents a shorter lithium transportation route that contributes to the high‐rate capabilities of the graphite electrode in ether‐based electrolytes. Moreover, we find that the ionic conductivity of the ether‐based electrolyte is 4.94 mS cm^−1^, which value is less than 9.41 mS cm^−1^ of the carbonate‐based electrolyte, further indicating the dominant influence of SEI on the rate performance of graphite than that of electrolyte properties in this case. Herein, we conclude that the different performances (i.e., rate capabilities) are mainly caused by the different SEI properties. Thus, these results offer an opportunity to pursue higher performance of LIBs, for instance, switching the electrolyte from ether‐based to carbonate‐based electrolyte after forming the aforementioned unique SEI on graphite in ether‐based electrolyte enables the battery to present high‐rate capabilities.

### 2.2 Benefits of Switching Electrolyte Interfacial Model

**Figure 4:**
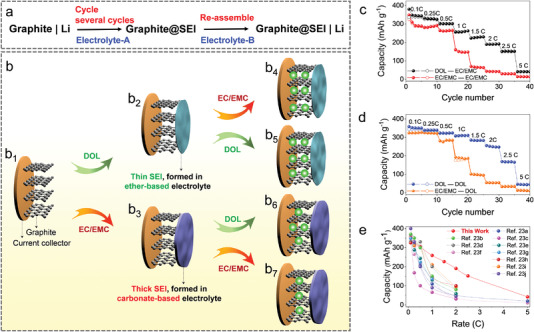
Benefits of switching electrolyte interfacial model. a) Illustration and b) Process of the exchange experiments. Comparative rate capabilities of c) the graphite@SEI (i.e., pre‐formed in the carbonate‐based electrolyte versus in ether‐based electrolyte) in the carbonate‐based electrolyte, and d) graphite@SEI (i.e., pre‐formed in carbonate‐based electrolyte versus in ether‐based electrolyte) in the ether‐based electrolyte. e) Comparison of the attained rate performance of graphite electrode by switching electrolyte with those reported before in carbonate‐based electrolytes.

Our concept was confirmed first by a newly designed exchange experiment (**Figure** **4**a,b). For example, the SEI was pre‐formed on graphite electrode (i.e., graphite@SEI) after being cycled in ether‐based or carbonate‐based electrolyte several times (Figure 4b_1_–b_3_), where such graphite@SEI was taken out and then reassembled in a new battery using another electrolyte (Figure 4b_4_–b_7_). There are four paths in this study as illustrated in Figure 4b_1_–b_7_. In detail, a thin graphite@SEI can be obtained in the ether‐based electrolyte, where such SEI can remain thin over the following cycles after being reassembled in the new battery using the ether‐based or carbonate‐based electrolyte (Figure 4b_1_ **→**b_2_ **→**b_4_,b_1_ **→**b_2_ **→**b_5_). In contrast, a thick graphite@SEI can be obtained in the carbonated‐based electrolyte, where such thick SEI can be maintained during cycling in the newly reassembled battery no matter using the carbonate‐based or the ether‐based electrolyte (Figure 4b_1_ **→**b_3_ **→**b_6_,b_1_ **→**b_3_ **→**b_7_). Thus, the two types of graphite@SEI endow the reassembled batteries with distinct capacities.

Firstly, when the carbonate‐based electrolyte was employed in the re‐assembled batteries, we find that the capacities of graphite@SEI that pre‐formed in the ether‐based electrolyte is 1.15, 1.72, 5.07, and 2.98 times higher than that pre‐formed in the carbonate‐based electrolyte at the rate of 0.25, 1, 2.5, and 5 C, respectively (Figure 4b_1_ **→**b_2_ **→**b_3_,b_1_ **→**b_3_ **→**b_7_,c). This result demonstrates that the SEI pre‐formed on graphite in the ether‐based electrolyte is beneficial to obtaining high‐rate capabilities in carbonate‐based electrolyte eventually. Besides, we find that the first Coulombic efficiency can be improved from 82.3% to 88.6% when the graphite@SEI electrode was re‐assembled in the new battery, i.e., from ether to carbonate‐based electrolyte. These results demonstrate that the pre‐formed thin SEI can be well maintained during the exchange experiment, and also such SEI can suppress the decomposition of carbonate‐based electrolyte on graphite effectively, enabling a high rate‐capability in the new battery.

The same phenomenon can be observed when the ether‐based electrolyte is used in the re‐assembled batteries. We find that the capacities of graphite@SEI that pre‐formed in the ether‐based electrolyte is 1.06, 1.68, 4.99, and 3.67 times higher than that pre‐formed in the carbonate‐based electrolyte at the rate of 0.25, 1, 2.5, and 5 C, respectively (Figure 4b_1_ **→**b_2_ **→**b_5_,b_1_ **→**b_3_ **→**b_6_,d). These comparative results verify that the SEI pre‐formed in the carbonate‐based electrolyte is too thick to obtain the high‐rate capability, so as to the following ether‐based electrolyte neither cannot improve the rate capabilities (Figure 4b_1_ **→**b_2_ **→**b_5_). This finding also manifests that the SEI is the dominant role to affect the rate capabilities of the graphite electrode in this case, which means the Li^+^ transportation within SEI is the determining factor of the Li^+^ (de‐)intercalation rate. In this part, by switching the electrolyte, high‐rate capabilities of the graphite electrode, such as 352, 329, 302, 259, 227, 191, 152, and 42 mAh g^−1^ those delivered at the rate of 0.1, 0.25, 0.5, 1, 1.5, 2, 2.5, and 5 C, respectively, can be obtained in the carbonate‐based electrolyte that has never been reported before (Figure 4e).^[^ ^23^ ^]^ This is a breakthrough because the merits of the specific thin SEI formed in the ether‐based electrolyte can function directly in the commercial carbonate‐based electrolyte, overcoming the issue of ether‐based electrolyte that can be decomposed easily at a high voltage operation (e.g., >4.0 V vs Li/Li^+^) in LIBs.

### 2.3 Electrolyte Interfacial Model Interpretation of SEI Formation

**Figure 5:**
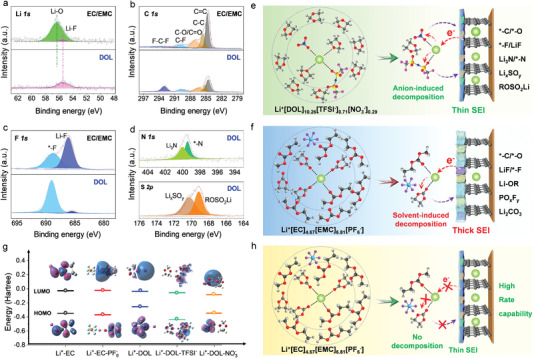
Formation mechanism of SEI. Comparative XPS spectra and fitting results of a) Li 1s, b) C 1s, and c) F 1s of SEI formed in ether‐based electrolyte versus carbonate‐based electrolyte. d) N 1s and S 2p of SEI formed in the ether‐based electrolyte. Schematic electrolyte decomposition behavior and SEI formation process on the graphite electrode in e) ether‐based electrolyte and f) carbonate‐based electrolyte. g) LUMO and HOMO energy of the Li^+^–EC, Li^+^–EC–PF_6_^−^, Li^+^–DOL, Li^+^–DOL–TFSI^−^, and Li^+^–DOL–NO_3_^−^ (insets are molecular orbital simulation snapshots of LUMO and HOMO). h) Schematic illustration of the SEI pre‐formed in the ether‐based electrolyte that mitigates carbonate electrolyte decomposition and also contributes to the high‐rate capabilities.

Investigations on the formation mechanism of SEI were carried out from a molecular insight both experimentally and computationally to analyze the compositions and structures of SEI from these two electrolytes, and accordingly, an electrolyte interfacial model was constructed to visually demonstrate the SEI formation process. The elemental differences of SEI compositions were studied by X‐ray photoelectron spectroscopy (XPS). Several salient features of the comparative SEI can be summarized: i) the amounts of Li^+^‐based species formed in the ether‐based electrolyte is much lower than that formed in the carbonate‐based electrolyte (**Figure** **5**a), and particularly the Li^+^‐based species formed in carbonate‐based electrolytes comprise a low proportion of Li^+^–F species (5.4%) and a much higher proportion of Li^+^–O species (95.6%), indicating a solvent‐dominant decomposition. These features of the Li^+^‐based species reveal that the solvent decomposition in ether‐based electrolytes is limited compared to that in the carbonate‐based electrolyte. ii) The amount of C‐based species formed in the ether‐based electrolyte is also much lower than that formed in the carbonate‐based electrolyte (Figure 5b). This result corroborates with the analysis in Li 1s, indicating the limited solvent decomposition in the ether‐based electrolyte. Besides, the proportion of C–F‐based species formed in the ether‐based electrolyte (29.5%, resulting from the LiTFSI decomposition) is much higher than 13.6% of that formed in the carbonate‐based electrolyte (i.e., resulting from LiPF_6_ decomposition), indicating a serious decomposition of LiTFSI compared to that of LiPF_6_. This result demonstrates that SEI formation in ether‐based electrolytes is mainly attributed to the decomposition of the lithium salt (i.e., LiTFSI, LiNO_3_ as discussed later), while the SEI species in carbonate‐based electrolytes come primarily from the decomposition of carbonate solvent. Moreover, comparative Li 1s and C 1s results show that the ether‐based electrolyte decomposition is limited, thereby enabling to form a thinner SEI layer as observed under TEM. iii) The F‐based SEI species formed in the ether‐based electrolyte is mainly composed of the organic compounds (i.e., *–F, 97.1%), while the amounts of inorganic compounds (Li^+^–F, 2.9%) is much less than 59.4% of that formed in the carbonate‐based electrolyte (Figure 5c). The thin organic species in SEI endows the graphite electrode with better flexibility to endure the volume variation during Li^+^ (de‐)intercalation more stably compared to that of inorganics, in turn enabling better cycling stability, as evidenced by CV curves in Figure 2d. iv) The N and S‐based species can be detected in SEI from the ether‐based electrolyte (Figure 5d). These species result from the decomposition of TFSI^−^ and NO_3_^−^, where the element of N and S could contribute to forming a more robust SEI and also assist to transport Li^+^ faster via the covalent bond.

The main components of SEI are summarized in Figure 5e,f, of which the formation mechanism is elucidated molecularly from the viewpoint of solvation structure and interfacial model. In the ether‐based electrolyte, i.e., Li^+^[DOL]_10.26_[TFSI^−^]_0.71_[NO_3_^−^]_0.29_ shown in Figure 5e, the NO_3_^−^ and TFSI^−^ anions have a high frequency to appear in the first solvation structure layer due to the low dielectric constant of DOL (*ε* = 6.79) and the weak interaction between DOL and Li^+^. Then, the anions can appear around Li^+^ during Li^+^ de‐solvation process and accept the electron from the electrode readily, giving rise to an anion‐dominant electrolyte decomposition on the graphite electrode. It also reveals why there is no Li^+^‐solvent (i.e., Li^+^‐DOL) co‐insertion into the graphite electrode observed in the ether‐based electrolyte, since the anion can weaken Li^+^–DOL interaction and then inhibit Li^+^–DOL co‐insertion into graphite. In contrast, in the carbonate‐based electrolyte, i.e., Li^+^[EC]_4.68_[EMC]_6.79_[PF_6_^−^] shown in Figure 5f, the PF_6_^−^ anion has a high frequency to appear in the second solvation structure layer due to the high dielectric constant of EC (*ε* = 89.8) and strong interaction between EC and Li^+^. Thus, EC can appear around Li^+^ during the Li^+^ de‐solvation process and accept the electron from the electrode readily, giving rise to a solvent‐dominant electrolyte decomposition on the graphite electrode.

The simulation results also corroborate our conjecture. The calculated highest‐occupied molecular orbital (HOMO) and lowest‐unoccupied molecular orbital (LUMO) of Li^+^‐solvent and Li^+^‐solvent‐anion complexes are shown in Figure 5g. We find that the LUMO values of Li^+^–EC–PF_6_^−^ complex (−0.023 Hartree) increased compared to Li^+^–EC (−0.027 Hartree), while the LUMO value of Li^+^‐DOL‐anion complex (i.e., −0.055 Hartree for TFSI^−^, −0.084 Hartree for NO_3_^−^) is lower than that of Li^+^–DOL (−0.036 Hartree). It shows that the solvent in the carbonate‐based electrolyte is easier to be decomposed by reduction, while the anion (i.e., TFSI^−^, NO_3_^−^) in the ether‐based electrolyte is easier to get electrons to be decomposed. The Li_3_N and *−N species shown in the N 1s spectrum of XPS results in Figure 5d can be attributed to the decomposition product of NO_3_^−^ and TFSI^−^, respectively, which can also corroborate the rationality of our analysis in simulation. Moreover, further decomposition of the ether‐based electrolyte can be inhibited once part of the ether solvent (such as DOL) together with the anions is reduced and polymerized into a thin SEI film,^[^ ^24^ ^]^ enabling high‐rate capabilities. In contrast, the electrolyte decomposition is more severe in the carbonate‐based electrolyte, in turn forming a thick SEI and giving rise to low‐rate capabilities. In this way, by switching the electrolyte interfacial model from ether‐based to carbonate‐based electrolyte (Figure 5h), the decomposition of the carbonate‐based electrolyte can be mitigated once such a thin SEI is pre‐formed on the graphite electrode in the ether‐based electrolyte, since the electron‐donating capability of graphite electrode is lowered by the thin SEI, in turn improving the stability of the carbonate‐based electrolyte and the rate capabilities of graphite electrode.

Besides the reasonable interpretation of SEI by our interfacial model, an insightful explanation can be concluded further for the varied graphite performance when the LiNO_3_ was absent or DME solvent was added to the DOL‐based electrolyte. In the electrolyte without LiNO_3_, we find that the Li^+^–DOL and also the Li^+^–TFSI^−^ interaction were increased slightly, as judged by the blue shift of the stretching vibration of C–O in DOL (i.e., from 939.9/962.8 cm^−1^ to 940.1/963.1 cm^−1^) and S–N–S in TFSI^−^ (i.e., from 742.1/746.9 cm^−1^ to 742.8/747.5 cm^−1^), respectively (Figure S5a,b, Supporting Information). All Raman results re‐confirm that the NO_3_^−^ can participate in the solvation structure and weaken the Li^+^–DOL interaction, where the Li^+^–TFSI^−^ interaction can be also weakened slightly. In this way, in the absence of LiNO_3_ (i.e., 1.0 m LiTFSI in DOL, Li^+^[DOL]_14.37_[TFSI^−^]), the Li^+^ is hard to be de‐solvated readily in the interfacial model due to the increased Li^+^–DOL interaction, then leading to a low initial Coulombic efficiency (i.e., 71.7%, because of the aggravated Li^+^–DOL decomposition) and low rate capabilities (Figure S5c, Supporting Information). The constructed interfacial model can not only interpret the poor graphite performance in the absence of LiNO_3_ but also can show the reason for the good graphite performance in the presence of LiNO_3_ (Figure S5d, Supporting Information). While in the electrolyte with the introduction of DME solvent (i.e., 1.0 m LiTFSI, 0.4 m LiNO_3_ in DOL/DME, Li^+^[DME]_4.37_[DOL]_6.53_[TFSI^−^]_0.71_[NO_3_^−^]_0.29_), the strong coordination ability of DME to Li^+^ in the interfacial model can lead to a serious Li^+^–DME co‐insertion within graphite, thereby causing graphite exfoliation (Figure S5e, Supporting Information). Besides, our interfacial model can also interpret from a different viewpoint why the concentrated ether‐based electrolyte (e.g., 5.0 m LiTFSI in DOL, Li^+^[DOL]_2.87_[TFSI^−^]) can become compatible with the graphite.^[^ ^21^ ^]^ This is because the Li^+^–DOL interaction can be weakened effectively by the TFSI^−^ anion in the interfacial model due to the insufficiency of DOL solvent (Figure S5f, Supporting Information), thereby allowing a reversible Li^+^ (de‐)intercalation. In this section, we presented an electrolyte interfacial model to interpret the formation mechanism of SEI and also the reason for the varied graphite performance in different electrolytes from a molecular scale, based on which we can switch the electrolyte effectively for greater performances.

### 2.4 Fast‐Charging Rate LIBs Applications

**Figure 6:**
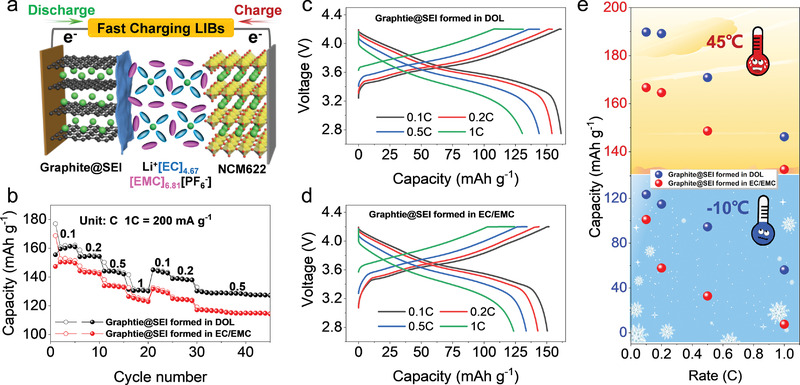
Fast‐charging rate LIBs applications. a) Schematic configuration of the LIBs composed of graphite@SEI anode and NCM622 cathode rate performance. Comparative rate capabilities b) and typical voltage versus capacity profiles c,d) of LIBs using the graphite@SEI (SEI formed in ether‐based versus carbonate‐based electrolyte) in the carbonate‐based electrolyte. e) Comparative rate performance of LIBs at high and low temperatures.

The advantages of SEI pre‐formed in ether‐based electrolyte were further verified in LIBs, in which the graphite@SEI anode was paired with the lithium layered metal oxide (e.g., LiNi_0.6_Co_0.2_Mn_0.2_O_2_) cathode to assemble the graphite@SEI | NCM622 full battery (**Figure** **6**a). We find that the rate capabilities can be significantly improved by switching the electrolyte interfacial model. For example, a high capacity of 161, 154, 143, and 130 mAh g^−1^ can be delivered at the (dis‐)charging rate of 0.1, 0.2, 0.5, and 1 C using graphite@SEI formed in the ether‐based electrolyte which is much higher than 150, 142, 133, and 123 mAh g^−1^ when the graphite anode was pre‐coated with the SEI in the carbonate‐based electrolyte (Figure 6b). Note that the increased rate capacities in the ratio of 7.3%, 8.4%, 7.5%, and 5.6% are already much improved in LIBs design,^[^ ^25^ ^]^ since the capacity of most cathodes is less than 200 mAh g^−1^ and hard to be improved further.^[^ ^26^ ^]^ Besides, the polarization of the (dis‐)charge curves of the battery using graphite@SEI formed in the ether‐based electrolyte is also much lower than that using graphite@SEI formed in the carbonate‐based electrolyte (Figure 6c,d), which is conformably attributed to the thin SEI with specific components formed in the ether‐based electrolyte that enables faster Li^+^ transportation and a minor electrolyte decomposition.

Furthermore, the merit of using SEI pre‐formed in the ether‐based electrolyte applies to the full battery operating at a wide‐range temperature from 45 to −10 °C. A better rate performance can also be obtained when the SEI was pre‐formed in the ether‐based electrolyte (Figure 6e), of which high capacities of 190, 189, 171, 146 mAh g^−1^ and 124, 115, 95, 56 mAh g^−1^ present at the rate of 0.1, 0.2, 0.5, and 1 C, at 45 and −10 °C respectively. These values are much higher than 167, 165, 149, 133 mAh g^−1^ and 101, 58, 33, 8 mAh g^−1^ when the SEI was pre‐formed in the carbonate‐based electrolyte. These results show the opportunity of pursuing the fast‐charging LIBs by switching the electrolyte interfacial model since the graphite electrode with pre‐formed SEI in the ether‐based electrolyte can cycle well in carbonate‐based electrolyte and also show high‐rate capabilities. To be noted, the ether‐based electrolyte cannot be used directly in LIBs since it is easy to be decomposed at the high voltage while the carbonate‐based electrolyte can remain stable. Moreover, the LiTFSI cannot be used as the lithium salt directly in the carbonate‐based electrolyte since it has a severe corrosion capability to the Al foil on the cathode side, causing a severe capacity decay (Figure S6a,b, Supporting Information). Thus, although the Li^+^ de‐solvation and the Li^+^ transportation rate penetrating through the SEI have both been considered to be the determinant step for designing the fast‐charging batteries both,^[^ ^27^ ^]^ herein the uniqueness of the pre‐formed thin SEI is the main factor for the obtained greater performance. Besides, the unique SEI can enhance the Li^+^ transportation rate and suppress the electrolyte decomposition, thereby improving the high‐temperature stability. By designing or employing a stable weakly solvent, we believe that the weakly solvated structure can weaken the Li^+^ solvent interaction further, speed up the de‐solvation process, and improve the fast‐charging and low‐temperature discharge capacity. In this section, we successfully applied the strategy of switching electrolytes to design the fast‐charging and wide‐temperature LIBs, showing the feasibility of this approach to design LIBs. Promoted by this concept, we believe that the economy and practicality can be much improved if the specific SEI can be achieved in the battery formation stage by designing and employing a specific electrolyte.

## 3 Conclusion

An innovative concept of switching electrolyte interfacial model to tune SEI properties was presented, by which the advantages of different electrolytes can be combined into one battery system readily. High‐rate capabilities that had never been reported before in the carbonate‐based electrolyte system were obtained using a graphite electrode with a pre‐formed unique and thin SEI in the ether‐based electrolyte, enabling to design of fast charging and wide‐temperature lithium‐ion batteries. We presented a molecular interfacial model to interpret the formation mechanism for the SEI, in which the conformation and electrochemical stabilities of Li^+^‐solvent‐anion were introduced to demonstrate the difference in SEI formation and the varied electrode performances in different electrolytes. We believe such a switching strategy and understanding of the SEI formation mechanism offers a new opportunity to design SEI and batteries with greater performances, such as better rate performance and longer lifespan.

## Conflict of Interest

The authors declare no conflict of interest.

## Supporting information

Supporting Information

## Data Availability

Research data are not shared.
