# Supplementary material for: Switching Electrolyte Interfacial Model to Engineer Solid Electrolyte Interface for Fast Charging and Wide‐Temperature Lithium‐Ion Batteries
Source: Adv Sci (Weinh). 2022 Jul 17;9(26):2201893. doi: 10.1002/advs.202201893 (PMC9475531; doi:10.1002/advs.202201893)
Supplement: Supplementary file 1 — Supporting Information [file ADVS-9-2201893-s001.pdf]

ADVANCED SCIENCE
Open Access

Supporting Information

for *Adv. Sci.*, DOI 10.1002/advs.202201893

Switching Electrolyte Interfacial Model to Engineer Solid Electrolyte Interface for Fast Charging and Wide-Temperature Lithium-Ion Batteries

Gang Liu, Zhen Cao, Peng Wang, Zheng Ma, Yeguo Zou, Qujiang Sun, Haoran Cheng, Luigi Cavallo, Shiyou Li, Qian Li* and Jun Ming**

Supporting Information

Switching Electrolyte Interfacial Model to Engineer Solid Electrolyte Interface for Fast Charging and Wide-Temperature Lithium-Ion Batteries

Gang Liu, Zhen Cao, Peng Wang, Zheng Ma, Yeguo Zou, Qujiang Sun, Haoran Cheng, Luigi Cavallo, Shiyou Li, Qian Li,* Jun Ming**

G. Liu, Y. G. Zou, Dr. Q.Sun, Dr. Z. Ma, H. Cheng, Dr. Q. Li, Prof. J. Ming
State Key Laboratory of Rare Earth Resource Utilization
Changchun Institute of Applied Chemistry, Chinese Academy of Sciences
Changchun, 130022, P. R. China
E-mail: zheng.ma@ciac.ac.cn; qianli@ciac.ac.cn.

G. Liu, Y. G. Zou, H. Cheng, Prof. J. Ming
University of Science and Technology of China
Hefei, 230026, P. R. China
E-mail: jun.ming@ciac.ac.cn.

Dr. Z. Cao, Prof. L. Cavallo,
Physical Science and Engineering Division (PSE)
King Abdullah University of Science and Technology (KAUST)
Thuwal 23955-6900, Saudi Arabia

Dr. P. Wang, Prof. S. Li
School of Petrochemical Technology, Lanzhou University of Technology
Lanzhou 730050, P.R. China

Keywords: lithium-ion battery, electrolyte solvation structure, solid electrolyte interface, fast charging, wide-temperature

Experimental and characterizations

Materials. The chemicals of Lithium bis(trifluoromethanesulfonyl)imide (LiTFSI), lithium hexafluorophosphate ($LiPF_6$), lithium nitrate ($LiNO_3$), ethylene carbonate (EC), ethyl methyl carbonate (EMC), and 1,3-Dioxolane (DOL) were purchased from DodoChem. The NCM622 was provided by the Huzhou Kunlun Power Battery Materials Co. Ltd. The graphite was purchased from Shanghai Shanshan Tech Co. Ltd, China.

Electrolyte preparation. All reagents were used directly without further purification. The stoichiometric ratio of lithium salt and solvent was calculated based on the molar concentration and then used to prepare the targeted electrolyte. Typically, the solvent was added to the brown bottle first, and then the lithium salt was then added under stirring to form a clear electrolyte solution. The entire process was handled carefully in the argon-filled glovebox, where the moisture and oxygen contents are controlled at about 0.5 ppm. The ionic conductivity was measured with a conductivity meter (Five Easy PlusTM-FE38, Mettler Toledo Co., Ltd) at room temperature.

Electrode preparation. The NCM powders, conductive materials (3.5 wt.% C45 and 1.5 wt.% KS-6), and poly (vinylidene fluoride) (PVDF) were mixed with the weight ratio of 92:5:3 in N-methyl pyrrolidinone (NMP). While for graphite anodes, the graphite powders, conductive carbon (SP), carboxymethyl cellulose (CMC), and styrene-butadiene rubber (SBR) were mixed with the weight ratio of 94.5:1.5:1.5:2.5 in water. The mixtures were milled using a Hasai planetary mixer for 5 min. Then, the uniform slurry was coated on the aluminum and copper foil, respectively. Finally, the NCM and graphite electrodes were dried at 120 °C and 80 °C in vacuum for 10 h, respectively. The mass loadings of the cathode and anode were about 8 mg cm^{-2} and 5.8 mg cm^{-2}, respectively.

Electrochemical measurements. All batteries were assembled using the *2032*-type coin cell and disassembled in an argon-filled glovebox. The graphite electrode performance was tested in the Li | graphite half-cell, in which the ether-based electrolyte (i.e., 1.0 M LiTFSI, 0.4 M $LiNO_3$ in DOL) or carbonate-based electrolyte (i.e., 1.0 M $LiPF_6$ in EC/EMC (v/v, 3/7)) was used. For convenience, we label the carbonate-based electrolyte as EC/EMC and the ether-based electrolyte as DOL. The cut-off voltage was set at 0.01-3.0 V. The graphite@SEI electrode was obtained as below. The Li | graphite half-cell was cycled for three cycles, where the graphite@SEI was formed first and then taken out after disassembling the cell. The graphite@SEI was washed and dried carefully, which was then tested using different kinds of

electrolytes (i.e., switching the electrolyte), for example, from the ether-based electrolyte to carbonate-based electrolyte, or from the carbonate-based electrolyte to ether-based electrolyte.

Besides, the graphite@SEI | NCM622 full battery was assembled and tested, in which the N/P ratio (i.e., the total capacity ratio of graphite@SEI/NCM622) was controlled at around 1.2 and the carbonate-based electrolyte was used. The cut-off voltage was set at 2.75-4.25 V. The high-temperature cycling test of the batteries was conducted in a constant temperature oven at 45 °C. The low-temperature cycling test of the batteries was performed in a low-temperature incubator at minus -10°C, which were pre-cycled three times at room temperature (25°C) before the low-temperature cycling. All galvanostatic charge/discharge curves were recorded by the Neware instrument. The electrochemical impedance spectroscopy (EIS) measurements were tested by the electrochemical station of *Bio-Lo*gic VMP3. The in-situ EIS test was performed on the entire discharge process, where the potential was decreased from the open-circuit voltage (OCV) to 0.01 V gradually by the interval of 5 mV. In each test, the sinusoidal AC perturbation of 5 mV over the frequency range from 100 kHz to 10 MHz was adopted. The GITT method was used to measure D_{Li+} (testing at 0.1 A g^{-1}, pulse time 20 min, relaxation time 30 min).

Materials characterizations. The morphology of electrodes was observed by scanning electron microscopy (SEM, Hitachi S-4800), while their structural characteristics were observed via transmission electron microscopy (TEM, FEI Tecnai G2 F20). The XPS spectra of the graphite and NCM electrodes were measured by X-ray photoelectron spectroscopy (XPS, ESCALABMKLL) with Al Kα radiation, which emits 1.4866 keV X-ray with the corresponding wavelength of 8.53 Å.

Theoretical simulation. The binding energy and molecular orbitals were studied based on the gas phase calculations and the implicit solvent models using the Gaussian09 package.

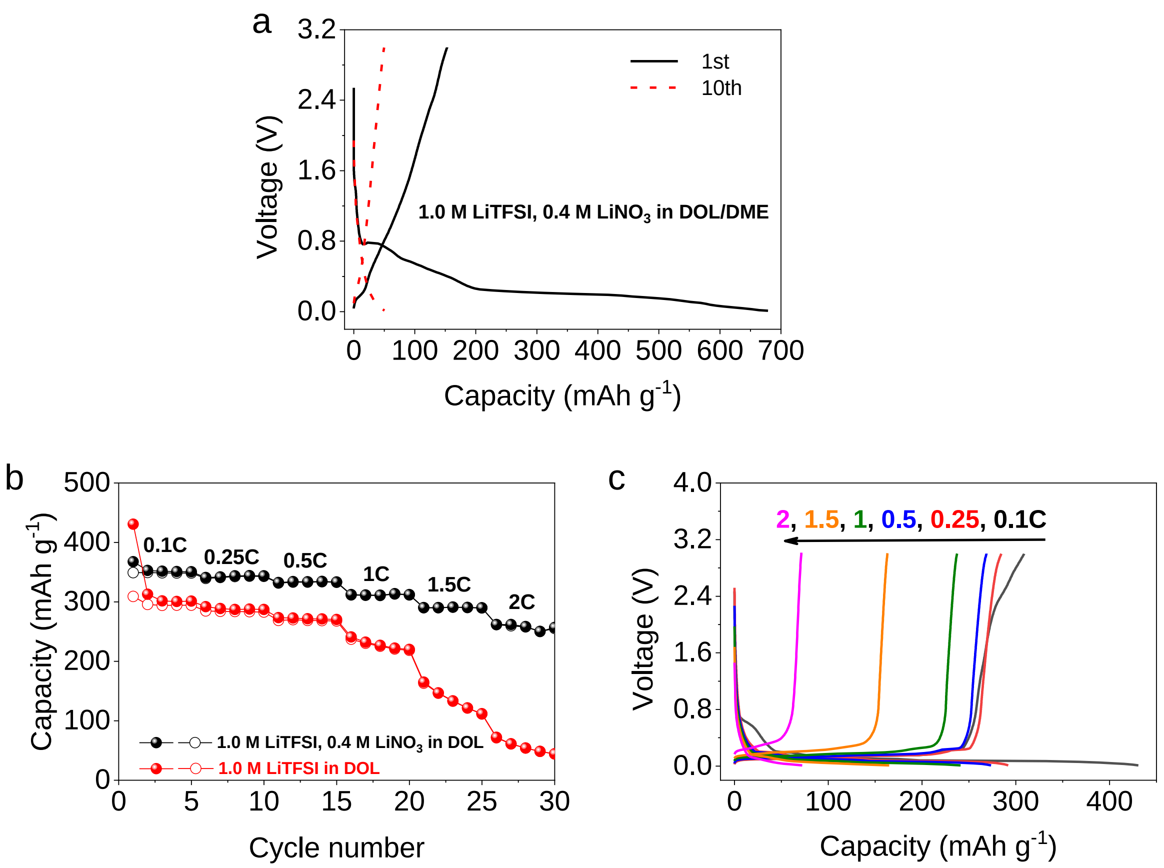

Figure S1. Graphite performance in different electrolytes. (a) Typical voltage versus capacity profiles in 1.0 M LiTFSI, 0.4 M $LiNO_3$ in DOL/DME, (b) Comparison of the rate capabilities in the DOL-based electrolyte with and without $LiNO_3$. (c) Typical voltage versus capacity profiles under different rates in 1.0 M LiTFSI in DOL without $LiNO_3$.

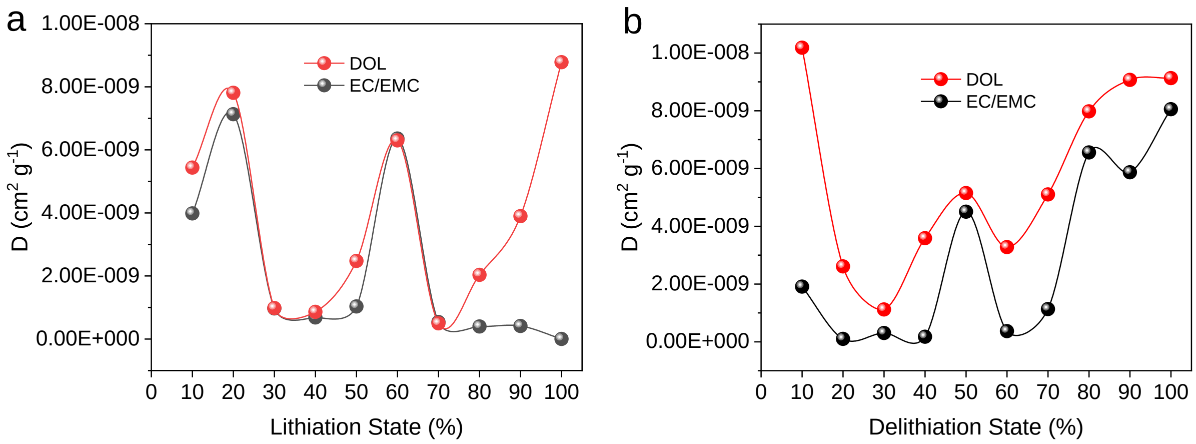

Figure S2. Li^+ diffusion coefficient (D_{Li+}) of graphite electrode during the discharge (a) and charge (b) processes in ether-based electrolyte vs. carbonate-based electrolyte.

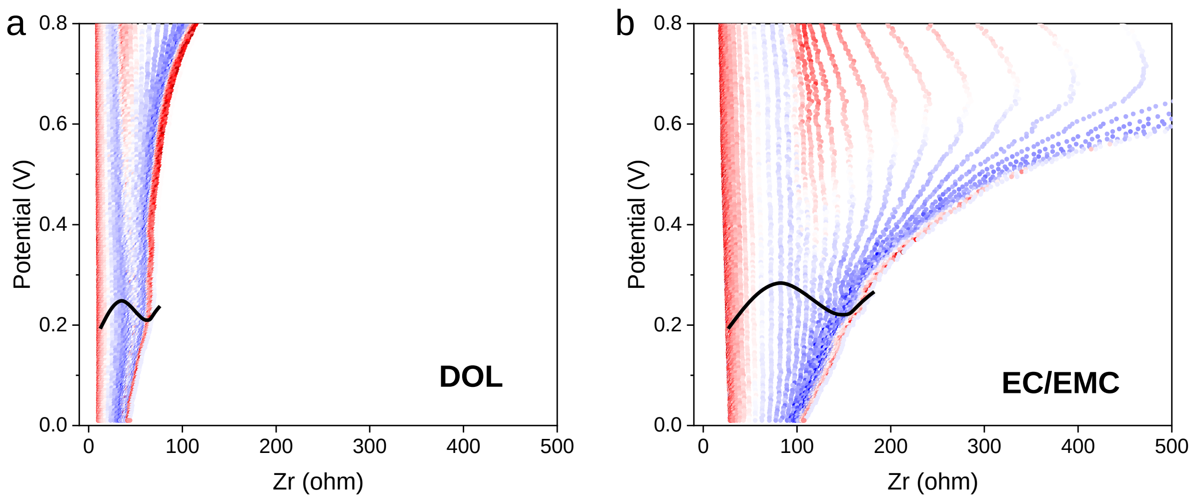

Figure S3. Partially enlarged region of *in-situ* EIS for the graphite electrode in the electrolyte of (a) 1.0 M LiTFSI in DOL and (b) 1.0 $LiPF_6$ in EC/EMC.

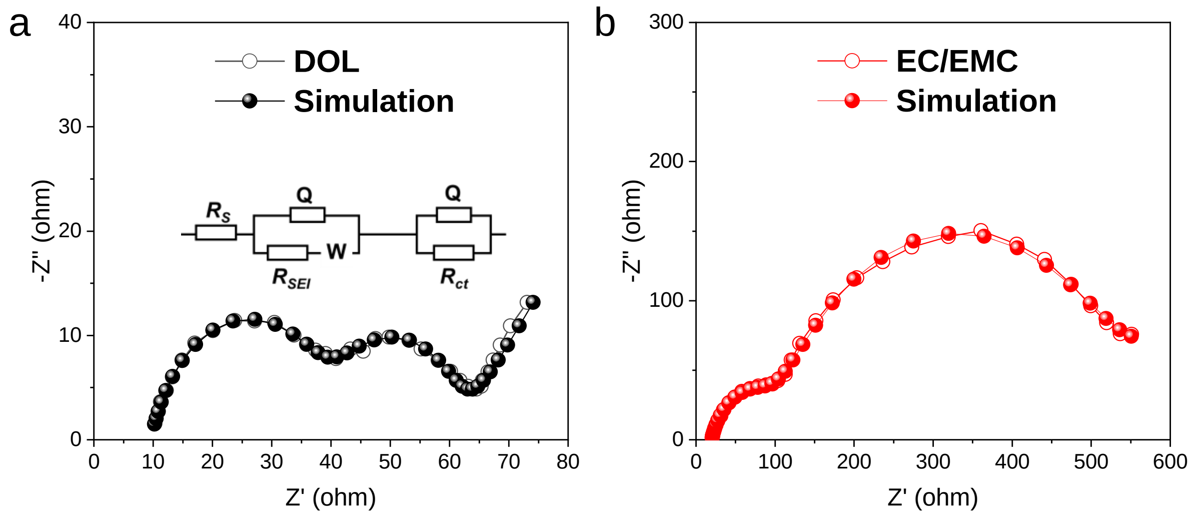

Figure S4. Electrochemical impedance spectroscopy (EIS) of the graphite electrode that discharged in (a) ether-based electrolyte and (b) carbonate-based electrolyte. The EIS curve consists of a high-frequency semicircle (i.e., the impedance of the formed SEI, R_{SEI}), a mid-frequency semicircle (i.e., the charge transfer impedance, R_{ct}), and the low-frequency slash (i.e., Warburg impedance, W). Note that the EIS of the Li | graphite half-cell cell was measured at 50% state of charge (SOC).

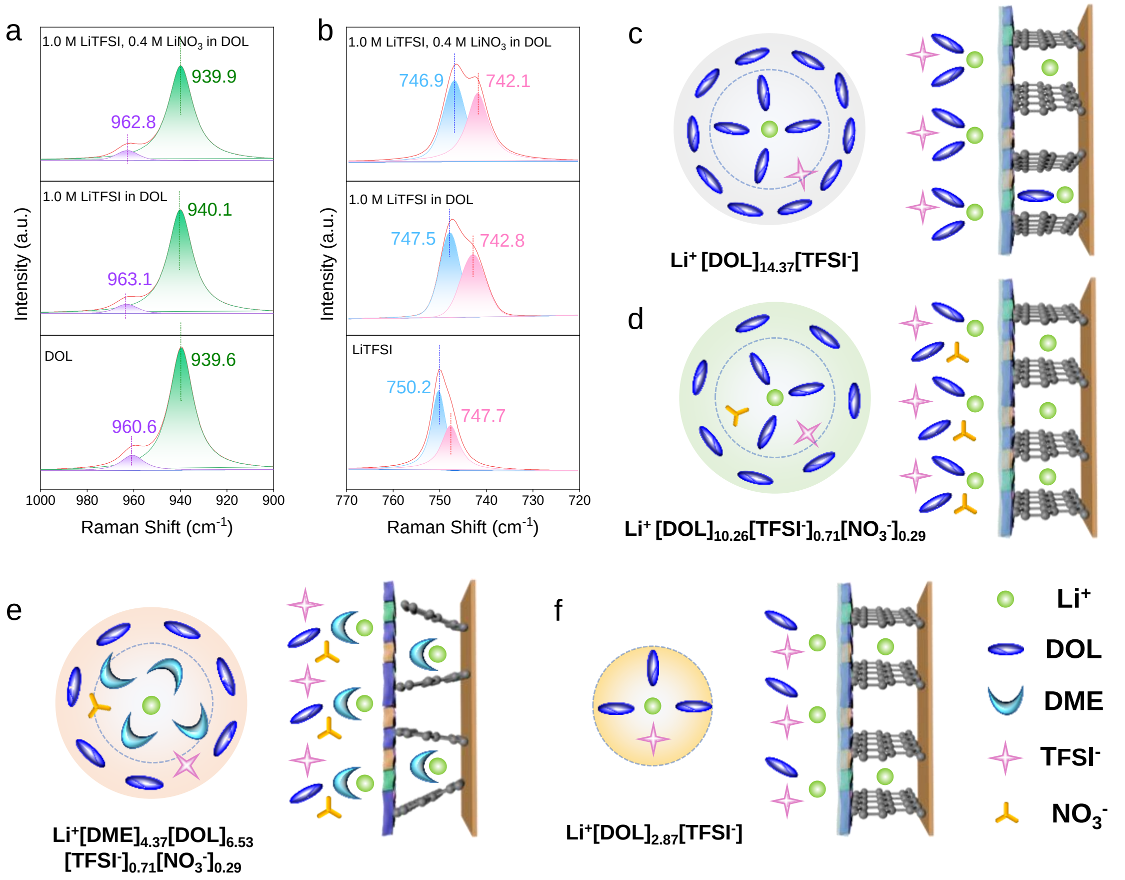

Figure S5. Characterization of electrolytes and their proposed solvation structure and interfacial model. Stretching vibration of (a) C-O in DOL and (b) S-N-S in $TFSI^-$ in the electrolyte with and without $LiNO_3$ in DOL-based electrolyte. Schematic illustration of the solvation structure and the derived interfacial model in the electrolyte of (c) 1.0 M LiTFSI in DOL, (d) 1.0 M LiTFSI, 0.4 M $LiNO_3$ in DOL, (e) 1.0 M LiTFSI, 0.4 M $LiNO_3$ in DOL/DME, (f) 5.0 M LiTFSI in DOL.

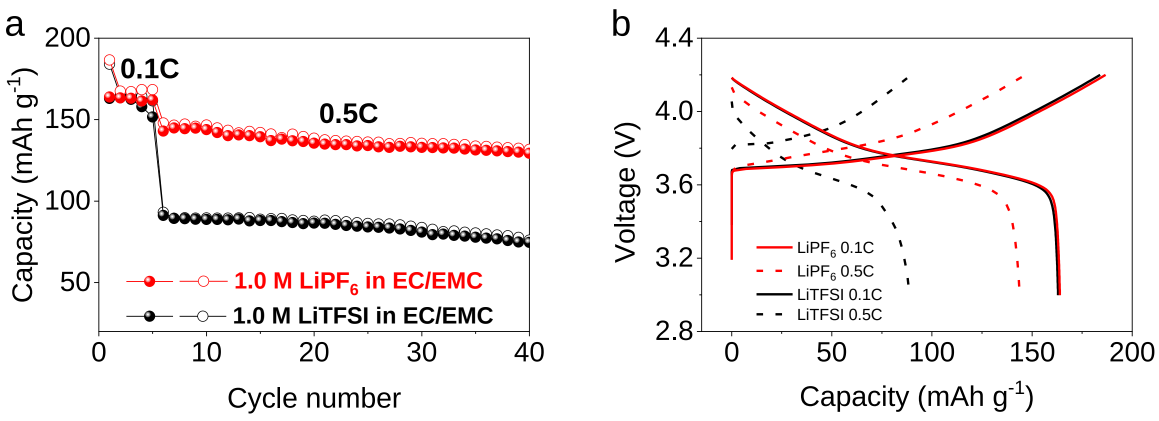

Figure S6. Electrochemical performance of NCM622 in different electrolytes. (a) Comparative rate capabilities and (b) typical voltage versus capacity profiles in the electrolyte of 1.0 M LiTFSI in EC/EMC and 1.0 M $LiPF_6$ in EC/EMC. The volumetric ratio of EC/EMC is controlled at 3/7.
